# Suppressing Sart1 to modulate macrophage polarization by siRNA-loaded liposomes: a promising therapeutic strategy for pulmonary fibrosis

**DOI:** 10.7150/thno.48152

**Published:** 2021-01-01

**Authors:** Ting Pan, Qing Zhou, Kang Miao, Lei Zhang, Guorao Wu, Jun Yu, Yongjian Xu, Weining Xiong, Yong Li, Yi Wang

**Affiliations:** 1 Department of Respiratory and Critical Care Medicine, Key Laboratory of Pulmonary Diseases of Health Ministry, Key Site of National Clinical Research Center for Respiratory Disease, Wuhan Clinical Medical Research Center for Chronic Airway Diseases, Tongji Hospital, Tongji Medical College, Huazhong University of Sciences and Technology, 1095 Jiefang Ave, Wuhan 430030, China.; 2 The Center for Biomedical Research, Tongji Hospital, Tongji Medical College, Huazhong University of Sciences and Technology, 1095 Jiefang Ave, Wuhan 430030, China.; 3 Department of Thoracic Surgery, Tongji Hospital, Tongji Medical College, Huazhong University of Sciences and Technology, 1095 Jiefang Ave, Wuhan 430030, China.; 4 Department of Respiratory Medicine, Shanghai Ninth People's Hospital, Shanghai Jiaotong University School of Medicine, 639 Zhizaoju Lu, Shanghai, 200011, China.

**Keywords:** Pulmonary fibrosis, Macrophage, siRNA, Sart1, Liposomes

## Abstract

Idiopathic pulmonary fibrosis (IPF) is a chronic and diffuse form of interstitial lung disease of unknown etiology with a fatal outcome. Although various strategies for IPF have been developed over the last few decades, no significant positive impact on the prognosis of IPF has been observed. According to the current paradigm, macrophages have been recognized to play a significant role in IPF pathogenesis. Here, we report a potential nanomedicine-based gene therapy for IPF based on regulate macrophage polarization.

**Method:** C57BL/6 mice were obtained and used to establish a bleomycin (BLM)-induced pulmonary fibrosis animal model, and *Sart1* siRNA-loaded liposomes were designed for *in vivo* experiment. The experimental animals were administered BLM intratracheally on day 0 and treated with *Sart1* siRNA on days 14 and 17. In the *in vitro* experiment, we further examined the function of Sart1 in macrophages.

**Results:** Our data indicated that the liposomes could passively target the fibrotic area in the lung and efficiently accumulate in macrophages. The suppression of Sart1 by siRNA-loaded liposomes significantly protected mice against BLM-induced lung injury and fibrosis, which was attributed to attenuated M2 macrophage infiltration in the lung.

**Conclusion:** Our study provides a valuable reference for modulating macrophage polarization and a promising strategy for the treatment of pulmonary fibrosis in clinical settings.

## Introduction

Idiopathic pulmonary fibrosis (IPF), a disease of unknown etiology, is characterized by the excessive deposition of extracellular matrix proteins and accumulation of fibroblasts and macrophages, which results in progressive scarring of alveolar-capillary units, eventually leading to gas exchange impairment and respiratory failure 1. Although pirfenidone and/or nintedanib treatment improves patient wellbeing, the prognosis of IPF remains poor, with 5-year mortality rates still ranging from 70 to 80% 2, 3. Consequently, the development of a novel effective treatment for IPF is urgently needed.

Macrophages, the most abundant innate immune cell type in normal lung tissue 4, are characterized by their plasticity and diversity. Depending on the local microenvironment, macrophages manifest two distinctive phenotypes, the classically activated phenotype (M1) and the alternatively activated phenotype (M2) 5. In general, M1 macrophages contribute to the host defense against pathogens by generating reactive nitric oxide (NO) *via* inducible nitric oxide synthase (iNOS) and releasing proinflammatory cytokines and chemokines such as IL-1β, IL-12, IL-23, CCL2 and TNF-α, while M2 macrophages have been shown to suppress inflammation and promote wound healing by producing cytokines and chemokines such as TGF-β, PDGF, CCL17 and CCL18 6, 7. According to the current paradigm, IPF patients and BLM-induced pulmonary fibrosis mouse model are infiltrated by predominantly M2 macrophages during the course of disease development and progression 8. Importantly, targeting the activation of M2 macrophages could be a viable therapeutic strategy for IPF 9, 10. Indeed, pirfenidone, an approved drug for IPF in clinical settings, exerts its antifibrotic effect in part by suppressing polarization to the M2 macrophage phenotype 11.

Gene therapeutic agents (e.g., siRNAs, mRNAs) are powerful tools to modulate macrophage function 12, 13. Nevertheless, viruses, a common gene vector, may cause a series of side effects, such as insertion mutagenesis and an inflammatory response, which makes viral vectors ineligible for *in vivo* therapeutic application 14. Over the past few decades, nanomedicines have emerged as potential platforms to overcome the pharmacokinetic obstacles associated with conventional medicines and have been shown to participate in the treatment of various diseases 15. The utilization of nanoparticles for nuclear acid delivery also shows enormous advantages in terms of excellent biocompatibility, high encapsulation efficiency, reduced gene degradation and enhanced targeting capability 16. In 2018, the first siRNA drug based on liposomes (patisiran) was approved by the US Food and Drug Administration, which moved gene therapy from concept to clinical application 17. Moreover, nanoparticles can be efficiently internalized by macrophages because macrophages are responsible for the phagocytosis of heterogeneous substances 15. Therefore, nanocarriers are well suited for macrophage-targeted drug delivery. However, the application of nanomedicine-based gene therapy to regulate macrophage polarization is rarely reported.

In the current study, we attempted to modulate macrophage polarization to alleviate pulmonary fibrosis by siRNA-loaded liposomes. Spliceosome associated factor 1 (SART1), an isoform-specific E3 ubiquitin ligase, is expressed in malignant proliferating tissue and has been recognized as involved in spliceosome assembly and cell division 18, 19. Recently, SART1 was shown to specifically degrade HIF-1α in an oxygen-independent manner 18, which was involved in the polarization of macrophages to the M2 program 20. Interestingly, notable overexpression of SART1 was observed in macrophages originating from IPF patients and mice with pulmonary fibrosis. Therefore, *Sart1* siRNA was selected as a potential target. In a mechanistic study, for the first time, we have used gain- and loss-of-function assays to verify that silencing Sart1 expression can repress M2 macrophage polarization by blocking the STAT6/PPAR-γ signaling axis. For efficient delivery of siRNA to macrophages in the lung, siRNA-loaded liposomes were raised. Impressively, *via* intratracheal injection, the liposomes could passively target the fibrotic area in the lung and accumulate in macrophages with high efficiency. An *in vivo* experiment demonstrated that suppression of Sart1 by siRNA-loaded liposomes significantly protected mice against BLM-induced lung injury and fibrosis, which was attributed to attenuated M2 macrophage infiltration in the lung. These data highlight the modulation of macrophage polarization by *Sart1* siRNA-loaded liposomes as a promising strategy for the treatment of pulmonary fibrosis in clinical settings.

## Methods

### Materials

Antibodies: Antibodies against fibronectin and collagen I were purchased from Cell Signaling Technology (MA, USA). Antibodies against Sart1, Arginase 1, β-actin, Gapdh and F4/80 were purchased from Proteintech (Hubei, China). Recombinant IL-4 was purchased from BioLegend (CA, USA). Antibodies against STAT6, p-STAT6 and PPAR-γ were obtained from Cell Signaling Technology (MA, USA).

Lipids: Cholesterol and DSPC were acquired from Sigma-Aldrich, Inc. (St. Louis, MO). mPEG2000-DMG (MW2660) was purchased from NOF Co., Ltd. (Kawasaki Japan). The cationic lipidoid C12-200 was generated through ring opening of epoxides by amine substrates with a previously reported method 21. Clodronate liposomes (anionic) were purchased from FormuMax (CA, USA).

Inhibitors: Inhibitors targeting STAT6 (AS1517499) and PPAR-γ (T0070907) were purchased from MedChemExpress (NJ, USA).

BLM was obtained from Hisun Pharmaceutical Co., Ltd (Zhejiang, China). An RT-PCR assay kit was supplied by Takara (Liaoning, China). A hydroxyproline assay kit was obtained from BioVision (CA, USA). Cell Counting Kit-8 was purchased from Dojindo Chemical technology Co. Ltd. (Shanghai, China).

### Human samples

Lung tissues were collected from patients with non-small cell lung cancer (NSCLC, n = 6) and patients with IPF (n = 8) in Tongji Hospital after informed consent was received. IPF was diagnosed according to the ATS/ERS consensus diagnostic criteria 22. The experiments were approved by the Human Assurance Committee of Tongji Hospital. Clinical data and the results of pulmonary function tests are provided in Table 1.

### RNA interference and plasmid transfection

Three siRNAs targeting mouse *Sart1* and a nontargeting control siRNA (named si-*Sart1*_1, si-*Sart1*_2, si-*Sart1*_3 and scramble siRNA, respectively) were purchased from RiboBio (Guangdong, China). The specific *Sart1*-targeted siRNA sequences were as follows: si-*Sart*1_1: 5'-AGA CCA AAC GGA GAG TGA A-3', si-*Sart*1_2: 5'-CCC AGA AGA CAC CGT ATA T-3', and si-*Sart1*_3: 5'-GCG AAC ACC ATC ACC AAA T-3'. The expression plasmid (pCMV-*Sart1* (GV657)) and NC plasmid were purchased from GeneChem (Shanghai, China). For transfection, Lipofectamine 3000 (Invitrogen, CA, USA) was used according to the manufacturer's instructions.

### Preparation and characterization of *Sart1* siRNA-loaded liposomes

Liposomes were raised as carriers to encapsulate siRNA. The siRNA was dissolved in citrate buffer (10 mM, pH = 3) and rapidly mixed with a lipid mixture by vortexing. The lipid mixture was composed of a lipidoid, cholesterol, DSPC and mPEG-DMG dissolved in ethanol at a molar ratio of 50:38.5:10:1.5. The unentrapped siRNA was removed by ultrafiltration centrifugation. The entrapment efficiency was measured by RiboGreen assay. The siRNA-loaded liposomes were used for *in vitro* and *in vivo* experiments after being diluted with PBS. The results of liposomes characterization (their hydrodynamic diameter, zeta potential, polydispersity and stability) are listed in Figure 2. These features were measured by dynamic light scattering (DLS) (Malvern Zetasizer Nano-ZS, UK). The morphology of the liposomes was investigated by transmission electron microscopy (TEM, Tecnai G2-20).

### *In vivo* biodistribution of the liposomes

Free DiR was added to the lipid mixture. DiR-loaded liposomes were prepared with the above method. Then, the liposomes were administered to pulmonary fibrosis model mice *via* intratracheal injection. The mice were then anesthetized and imaged at different time points (0 h, 6 h, 12 h, 24 h, 3 d, 6 d) by an *in vivo* imaging system (IVIS Lumina XR, Caliper, USA) (excitation: 748 nm, emission: 780 nm). After 6 d, the mice were sacrificed, and the organs were harvested for *ex vivo* fluorescence imaging.

### BLM-mediated induction of pulmonary fibrosis

Male C57BL/6 mice (8 weeks old) were obtained from Beijing Vital River Laboratory Animal Technology Co., Ltd. (Beijing, China). All animals were housed in a specific pathogen-free animal facility at Tongji Hospital under a 12:12 h light/dark photocycle and provided food and water ad libitum. All experimental procedures were approved by the Animal Care and Use Committee at Tongji Hospital.

In the therapeutic experiments, mice were divided into three groups: a (i) Control group treated with PBS, (ii) Control group treated with liposomes, (iii) BLM group, (iv) BLM+ liposomes group, (v) BLM + scramble siRNA-loaded liposomes group, and (vi) BLM + *Sart1* siRNA-loaded liposomes group. All the mice were anesthetized by intraperitoneal injection of pentobarbital sodium (70 mg/kg), after which 2.5 mg/kg BLM diluted in PBS or PBS alone was administered *via* intratracheal injection as reported 23. On days 14 and 17 after BLM induction, the mice were administered *Sart1* siRNA-loaded liposomes, scramble siRNA-loaded liposomes or liposomes (1 mg/kg) *via* intratracheal injection. Finally, the mice were sacrificed 21 days after BLM induction for analysis of pulmonary fibrosis.

In the macrophage depletion experiments, mice were divided into two groups: a (i) BLM + clodronate liposomes + scramble siRNA-loaded liposomes group and (ii) BLM+ clodronate liposomes + *Sart1* siRNA-loaded liposomes group. Clodronate liposomes (15 mg/kg were administered intratracheally on day -1 and day 12 after BLM treatment. The scramble siRNA-loaded liposomes and *Sart1* siRNA-loaded liposomes were administered as above described.

### Histopathological analysis

The left lungs were inflated with 4% neutral buffered paraformaldehyde and then fixed in the above fluid for 24 h at room temperature, followed by paraffin embedding. The lung tissues were sliced into 5 µm sections and stained with hematoxylin and eosin (H&E) 24, Sirius red and Masson's trichrome as previously reported 25, 26. The severity of pulmonary fibrosis was scored on a scale from 0 to 8 based on the Ashcroft scale.

### Immunofluorescence analysis

For the immunofluorescence experiment, the slides were treated with anti-Sart1, anti-CD68, anti-F4/80 or anti-CD206 antibody at 4 °C overnight. All slides were washed with PBS before incubation with an Alexa 594-labeled anti-mouse and Alexa 488-conjugated anti-rabbit antibody (Invitrogen, CA, USA). After rinsed with PBS, the slides were exposed to DAPI for 5 min to stain nuclei. Then, the slides were analyzed under a fluorescence microscope.

### Flow cytometry analysis

On day 21, mice were administered DiO-labeled liposomes *via* intratracheal injection and sacrificed after 24 h. The lungs of the mice were minced into a homogenous paste and treated with 3 ml of 1 mg/ml collagenase 1 diluted in HBSS. After incubation in a 15 ml centrifuge tube for 1.5 h at 37 °C in a table concentrator, the cell suspensions were passed through a 100 µm Falcon nylon cell strainer (Corning, NY, USA) and resuspended in stain buffer (PBS containing 2% FBS). Antibodies against F4/80-VioBlue, CD11c-PE and CD11b-APC were obtained from BD Biosciences Pharmingen. Staining of 100 µl cell suspensions containing 1×10^6^ cells was performed in stain buffer at 4 °C for 30 min. The samples were washed twice prior to flow cytometry analysis.

### Quantitative analysis of hydroxyproline

The lung tissues were weighed and hydrolyzed as previously reported 27. A hydroxyproline kit from the Jiancheng Institute of Biotechnology (Jiangsu, China) was used to quantify the hydroxyproline content in the lung tissues according to the manufacturer's instructions.

### Quantitative Real-time PCR

Quantitative RT-PCR analysis was performed using SYBR Premix Ex Taq (Takara Liaoning, China) as previously reported 28. The primers for each target genes were as follow:* CD206* (5'- CTC TGT TCA GCT ATT GGA CGC-3', 5'- CGG AAT TTC TGG GAT TCA GCT TC -3'), *Ym1* (5'-GGG CAT ACC TTT ATC CTG AG-3', 5'-CCA CTG AAG TCA TCC ATG TC-3'),* Fizz1* (5'-TCC CAG TGA ATA CTG ATG AGA-3', 5'-CCA CTC TGG ATC TCC CAA GA-3'), *iNOS* (5'- AGG AAC CTA CCA GCT CAC TCT G-3', 5'- TTT CCT GTG CTG TGC TAC AGT T-3'), *Sart1* (5'- CTG ACT ACC TGC CCT ATG CG-3', 5'- AGA CGG AAG GAA TGT GGT CG-3'), and *Actb* (5'-TGA CGT TGA CAT CCG TAA AGA CC-3', 5'-CTC AGG AGG AGC AAT GAT CTT GA-3'). The relative expression levels of the different genes were quantified using the formula 2^-ΔΔct^ with *Actb* as the endogenous control and normalized to the control group.

### Western blot analysis

Culture cells and lung tissues were lysed with RIPA lysis buffer as previously described 27, 29, 30. Briefly, proteins were separated by 10% sodium dodecyl sulfate-polyacrylamide gel electrophoresis (SDS-PAGE) and then transferred onto polyvinylidene fluoride (PVDF) membranes. The membranes were incubated with primary antibody at 4 °C overnight. HRP-conjugated anti-mouse or anti-rabbit IgG was used as a secondary antibody for incubation for 1 h at room temperature. The reactive bands were detected by chemiluminescence (Advansta, CA, USA).

### Cell culture

Primary bone marrow-derived macrophages (BMDMs) were obtained by flushing mouse femurs and tibias and cultured in Roswell Park Memorial Institute (RPMI)-1640 medium with 10% fetal calf serum and a 1% solution of penicillin and streptomycin_._ Macrophage colony-stimulating factor (M-CSF) was added on days 1, 3, and 5 (50 ng/ml). All the cells were cultured at 37 °C in a humid atmosphere containing 5% CO_2_ and stimulated with 500 μg/ml LPS to promote M1 polarization or 10 ng/ml IL-4 to promote M2 polarization.

### Statistical analysis

Experimental results are expressed as the mean ± standard deviation. One-way ANOVA using GraphPad Prism 7.0 (GraphPad Software Inc., CA, USA) was performed for multiple group comparisons. Differences in means were considered statistically significant if *P* < 0.05.

## Results

### IPF is characterized by increased SART1 expression

**Figure 1:**
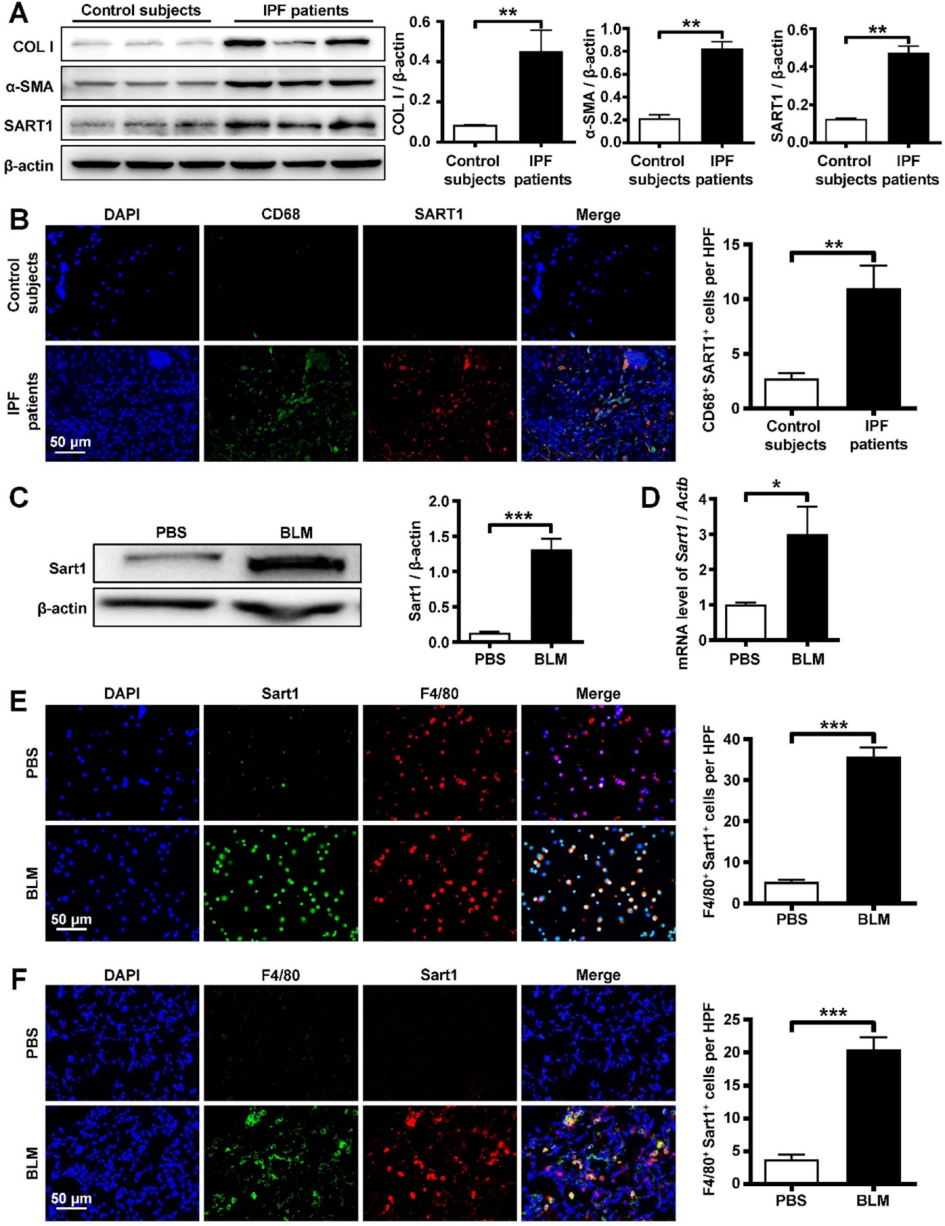
** Analysis of SART1 expression in patients with IPF and BLM-treated mice. A:** Western blot analysis of SART1, Col I and α-SMA expression in the lungs of IPF patients. Left panel: Representative Western blot. Right panel: Bar graph showing the mean data from all subjects analyzed in each group.** B:** Left panel: Representative results of immunostaining for SART1 and CD68 in lung sections from patients with IPF and control subjects. Right panel: Bar graph showing the mean data from the analyzed IPF patients. The images were taken at ×400 magnification. **C and D:** Western blot (C) and RT-PCR (D) analyses of Sart1 expression in the lungs of mice 21 days after BLM treatment. **E and F:** Representative results of coimmunostaining for Sart1 and F4/80 in BALF (E) and lung sections (F). The nuclei were stained blue by DAPI, and the images were taken at ×400 magnification. Each bar represents the mean ± SEM of 6 mice analyzed. *P < 0.05; **P < 0.01; ***P < 0.001. IPF: idiopathic pulmonary fibrosis; BLM: bleomycin; Col I: Collagen I; α-SMA: α-smooth muscle actin; HPF: high-power field.

To address the role of SART1 in IPF, we first examined SART1 expression in lung samples from IPF patients and control subjects. Interestingly, IPF patients manifested 3-fold greater SART1 expression in the lungs than control subjects, coupled with markedly higher expression of the fibrotic markers Collagen I and α-SMA (Figure 1A). Next, coimmunostaining was utilized to determine the cellular localization of SART1. As shown in Figure 1B, abundant SART1 was observed in the IPF patient lung sections, which was consistent with the Western blot results. Moreover, it was interesting to find that the majority of SART1 was expressed in IPF patient pulmonary macrophages (CD68^+^ cells, green).

To confirm the above results, pulmonary fibrosis model mice were developed by BLM induction, and lung samples were collected for further investigation. Similarly, Western blot and RT-PCR analyses revealed extremely increased Sart1 expression in the lungs of pulmonary fibrosis-bearing mice compared to control mice (Figure 1C-D). BLM induced pulmonary fibrosis was characterized by significant M2 macrophages (CD206 positive) infiltration (Figure S1). To determine the pattern of Sart1 expression in lungs after BLM injection, immunofluorescent staining of the BALF (Figure 1E) and lung tissue (Figure 1F) derived from PBS and BLM-induced mice was employed. The results demonstrated that Sart1 was notably overexpressed in macrophages (F4/80^+^), which coincided exactly with the results obtained from human samples.

Collectively, both human and mouse experiments have shown that the remarkable overexpression of Sart1 emerged in lung macrophages during the pathogenic process of pulmonary fibrosis. These results suggest that Sart1 might play a critical role in macrophage polarization.

### Preparation and characterization of *Sart1* siRNA-loaded liposomes

**Figure 2:**
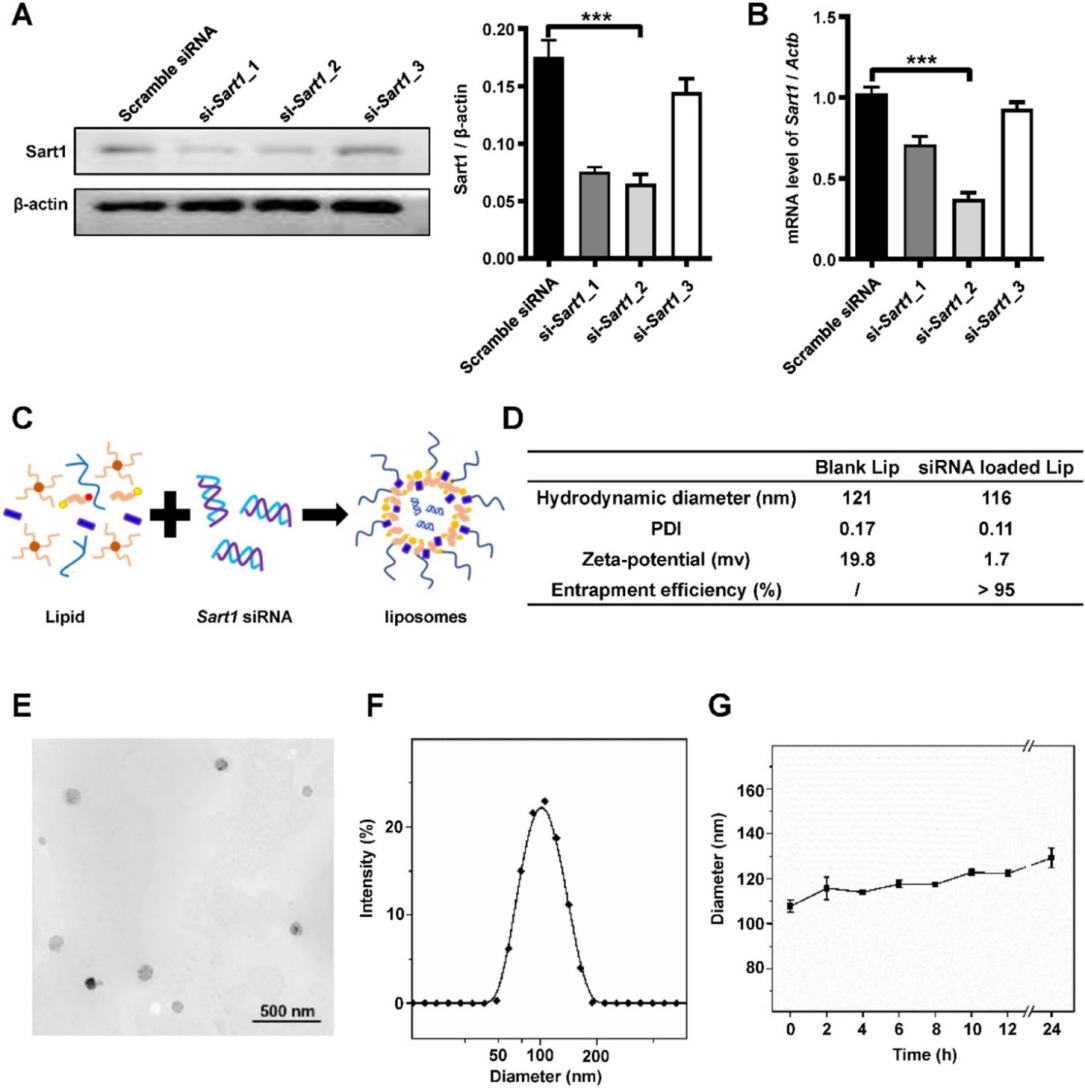
** Preparation and characterization of *Sart1* siRNA-loaded liposomes. A and B**: Western blot (A) and RT-PCR (B) analyses of the interference efficiency of *Sart1* siRNAs in RAW264.7 cells. **C:** Schematic diagram showing the preparation of *Sart1* siRNA-loaded liposomes. **D:** The hydrodynamic diameter, PDI and zeta potential of the liposomes (blank or siRNA-loaded) were measured by DLS. siRNA entrapment efficiency was measured by RiboGreen assay. **E:** Representative TEM image of siRNA-loaded liposomes. **F:** Hydrodynamic diameter distribution of siRNA-loaded liposomes. **G:** Colloid stability of siRNA-loaded liposomes in PBS. ***P < 0.001. PDI: polymer dispersity index; TEM: transmission electron microscopy; DSL: dynamic light scattering.

To verify the above hypothesis, three *Sart1* siRNA sequences were designed to repress the expression of Sart1 in macrophages. RAW264.7 cells were transfected with these three siRNAs with Lipofectamine 3000. Then, Western blot and RT-PCR analyses were conducted. The results revealed that si-*Sart1*_2 had the highest interference efficiency (Figure 2A-B). Therefore, in subsequent experiments, si-*Sart1*_2 was selected as the therapeutic siRNA.

*Sart1* siRNA-loaded liposomes were prepared for further *in vitro* and *in vivo* studies and characterized, and a simple and tested liposome formulation was adopted (Figure 2C). The final obtained liposomes were well dispersed in water, and their hydrodynamic diameter was approximately 116 nm, with the liposomes exhibiting a PDI of 0.11, as measured by DLS (Figure 2D-F). Due to the electronegative nature of siRNA, the zeta potential of siRNA-loaded liposomes was reduced (1.7 mV at 25 °C) compared to that of blank liposomes, which had a zeta potential of 19.8 mV (Figure 2D). A siRNA entrapment efficiency of over 95% was achieved for the prepared liposomes (Figure 2D). Additionally, a representative image was taken by transmission electron microscopy (Figure 2E) and showed that the nanoparticles possessed a spherical shape and manifested a uniform distribution (Figure 2E-F) with sustained stability over 24 hours (Figure 2G). The favorable biocompatibility of the siRNA-loaded liposomes was verified by CCK8 assay (Figure S2A). In addition, the *in vitro* transfection efficacy of the liposomes was as expected upon transfection with commercial Lipofectamine 3000 (Figure S2B).

### Suppression of Sart1 blunted the M2 program in macrophages by blocking the STAT6/PPAR-γ signaling axis

**Figure 3:**
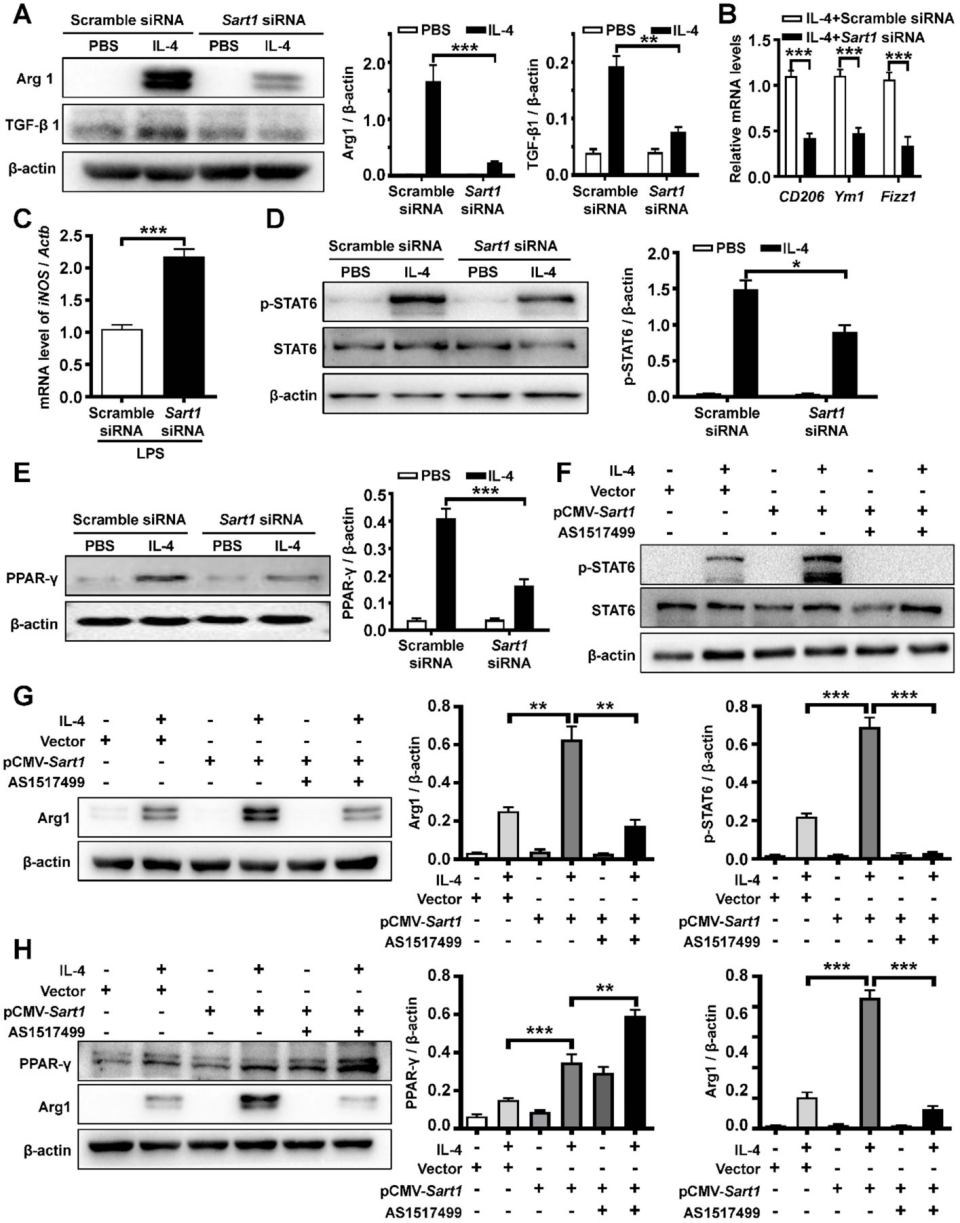
** The impact of Sart1 on IL-4-stimulated STAT6/PPAR-γ signaling in macrophages. A:** Arg 1 and TGF-β1 expression in BMDMs after IL-4 induction. Left panel: Representative Western blot. Right panel: Figures showing the data from 3 replicates.** B:** RT-PCR analysis of *CD206*, *Ym1* and *Fizz1* expression in BMDMs after IL-4 treatment. **C:** RT-PCR analysis of *iNOS* expression in BMDMs after LPS treatment. **D:** Knockdown of Sart1 attenuated IL-4-induced STAT6 phosphorylation. Left panel: Representative Western blot showing STAT6 and p-STAT6 at 1 h after IL-4 stimulation. Right panel: Bar graph showing the data from 3 replicates. **E:** Silencing of Sart1 blunted IL-4-induced PPAR-γ expression. Left panel: Representative Western blot showing PPAR-γ at 12 h after IL-4 stimulation. Right panel: Figures showing the data from 3 replicate.** F:** Overexpression of Sart1 enhanced IL-4-induced STAT6 phosphorylation. Up panel: Representative Western blot showing STAT6 and p-STAT6 at 1 h after IL-4 stimulation. Down panel: Figures showing the data from 3 replicates. **G:** Overexpression of Sart1 increased Arg 1 expression in BMDMs after IL-4 induction. Left panel: Representative Western blot. Right panel: Figures showing the data from 3 replicates. **H:** Effect of increased expression of Sart1 on PPAR-γ and Arg 1 expression. Left panel: Representative Western blot. Right panel: Figures showing the data from 3 replicates. *P < 0.05; **P < 0.01; ***P < 0.001. Arg1: Arginase-1; BMDM: bone marrow-derived macrophages; Fizz1: Found in inflammatory zone 1; iNOS: inducible nitric oxide synthase; STAT6: Signal transducer and activator of transcription 6; p-STAT6, phosphorylated STAT6; PPAR-γ: Peroxisome proliferator-activated receptor γ.

Based on the above results, we attempted to determine the impact of Sart1 on macrophage polarization and reveal the underlying mechanisms. BMDMs were generated and then subjected to IL-4 or LPS stimulation as previously described 31. Remarkably, Western blot analysis revealed significantly decreased M2 macrophage polarization in *Sart1* siRNA-loaded liposomes-treated BMDMs, as manifested by arginase-1 and TGF-β1 expression (Figure 3A), which was further corroborated by RT-PCR analysis of the expression of *CD206*, *Ym1* and *Fizz1*, other M2 macrophage markers (Figure 3B). Furthermore, blunting *Sart1* expression led to the upregulation of *iNOS* (a marker of M1 macrophages), indicating that Sart1 might also accelerate M1 macrophage polarization induced by LPS (Figure 3C).

Previous data have shown that the STAT6/PPAR-γ signaling pathway is critical for M2 macrophage polarization 32. Thus, we examined the effects of Sart1 on the STAT6/PPAR-γ signaling axis in BMDMs induced with IL-4. As expected, high levels of phosphorylated STAT6 (p-STAT6) were detected after 1 h of IL-4 stimulation (Figure 3D). However, compared to that in the group treated with scramble siRNA, the p-STAT6 level was substantially decreased in *Sart1* siRNA-treated BMDMs, suggesting that Sart1 might facilitate the polarization of M2 macrophages by enhancing STAT6 phosphorylation. In line with these results, the expression of PPAR-γ was also increased in BMDMs upon IL-4 stimulation, and the knockdown of Sart1 significantly inhibited the expression of PPAR-γ (Figure 3E).

For further verification, we next overexpressed Sart1 in BMDMs using the pCMV vector (pCMV-*Sart1*). As expected, the expression of Sart1 was enhanced following pCMV-*Sart1* transfection (Figure S3). Consistent with the above data, Western blot analysis revealed that the overexpression of Sart1 significantly enhanced the levels of p-STAT6 (Figure 3F) and PPAR-γ (Figure 3H), accompanied by the increased expression of arginase-1 (Figure 3G-H), indicating that Sart1 may increase adoption of the M2 program *via* STAT6/PPAR-γ signaling. To confirm this result, inhibitors of p-STAT6 (AS1517499) and PPAR-γ (T0070907) were applied following pCMV-*Sart1* transfection. Notably, the increase in arginase-1 induced by Sart1 was reversed.

Altogether, these results show that Sart1 accelerated M2 macrophage polarization by activating the STAT6/PPAR-γ signaling pathway, whereas *Sart1* siRNA-loaded liposomes dramatically attenuated M2 macrophage polarization by blocking the STAT6/PPAR-γ signaling axis.

### *In vivo* biodistribution of liposomes after intratracheal injection

**Figure 4:**
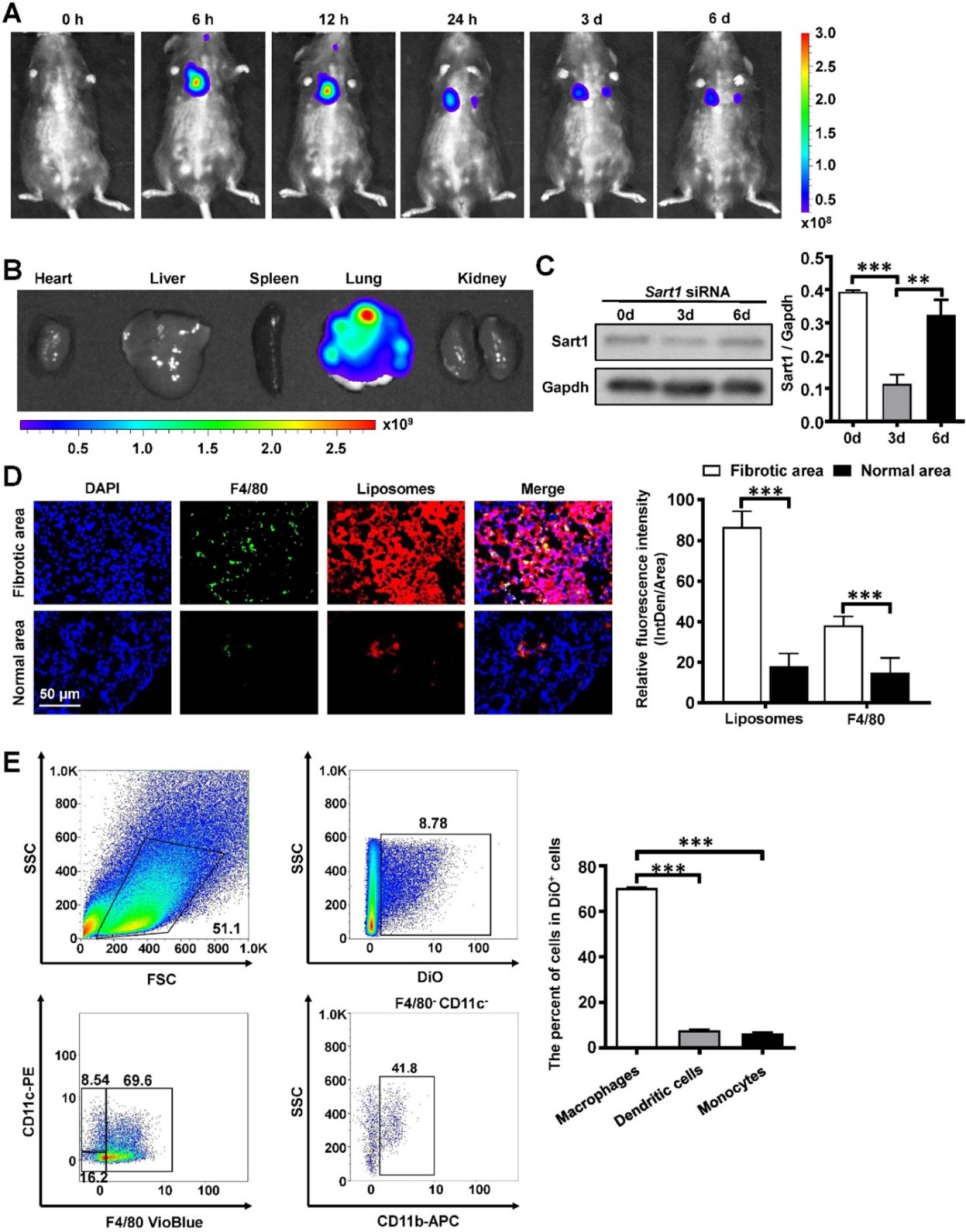
** Biodistribution of the liposomes after intratracheal injection. A:** Representative IVIS images of a mouse at different time points after the administration of DiR-labeled liposomes. **B:** *Ex vivo* fluorescence images of major organs from mice.** C:** Temporal changes in Sart1 expression in the lungs of transfected mice after BLM induction. **D:** Immunofluorescence image showing the biodistribution of DiR-labeled liposomes (red) and F4/80 (green) in the lungs of BLM-induced mice. The nuclei were stained blue by DAPI, and the images were taken at ×400 magnification.** E:** Flow cytometry analysis of the liposomes distribution in the lungs of BLM-induced mice. Six mice were included in each study group. **P < 0.01; ***P < 0.001.

For the following *in vivo* experiment, the biodistribution of the liposomes was explored in pulmonary fibrosis model mice. After intratracheal injection of the liposomes, pulmonary fibrosis model mice (n = 5) were surveyed and recorded by IVIS at different time points (0 h, 6 h, 12 h, 24 h, 3 d, 6 d). As shown in Figure 4A, the fluorescence signal was mainly concentrated in the lung and gradually decreased over time. An *ex vivo* image (Figure 4B) indicated that liposomes administered *via* intratracheal injection accumulated in the lung rather than in other organs. To further investigate the cellular localization of the liposomes, pulmonary slides were stained with immunofluorescence. Surprisingly, liposomes (red) were predominantly located in the fibrotic area (the area with crowded nuclei) of lung tissues and mainly overlapped with F4/80^+^ cells (green), indicating that liposomes could efficiently target pulmonary macrophages in a passive manner (Figure 4D). Additionally, the biodistribution of *Sart1* siRNA-loaded liposomes and Scramble siRNA-loaded liposomes showed no perceptible differences in the fibrotic area (Figure S4). To further confirm this result, flow cytometry was used with mice induced by BLM administered liposomes labeled with DiO. The majority of DiO^+^ cells were macrophages (F4/80^+^) but not dendritic cells or monocytes (Figure 4E). Some kinds of nanoparticles, such as graphene and porous silicon, can cause severe inflammatory responses in the lung 33, 34. To address this concern, normal mice were treated with liposomes, and pulmonary pathological sections were examined. The results demonstrated that almost no inflammatory infiltration was observed in the liposomes-treated group (Figure S5), indicating that the liposomes possess excellent biocompatibility *in vivo*. To determine an appropriate time interval for liposomes administration, we assessed the expression of Sart1 in the lungs after intratracheal injection of siRNA-loaded liposomes (Figure 4C). A significant decrease in Sart1 expression was noted on day 3 after the treatment, whereas the expression of Sart1 gradually increased and was restored on day 6 (Figure 4C). The Western blot results suggested that the optimal administration interval is 3 days, which prompted us to treat mice with liposomes at 14 d and 17 d after BLM induction.

Taken together, the above results verify that the liposomes selectively accumulated in the pulmonary fibrotic area, especially in macrophages, making them well suited for the treatment of pulmonary fibrosis.

### Suppression of Sart1 attenuated BLM-induced lung injury and fibrosis by targeting macrophages

**Figure 5:**
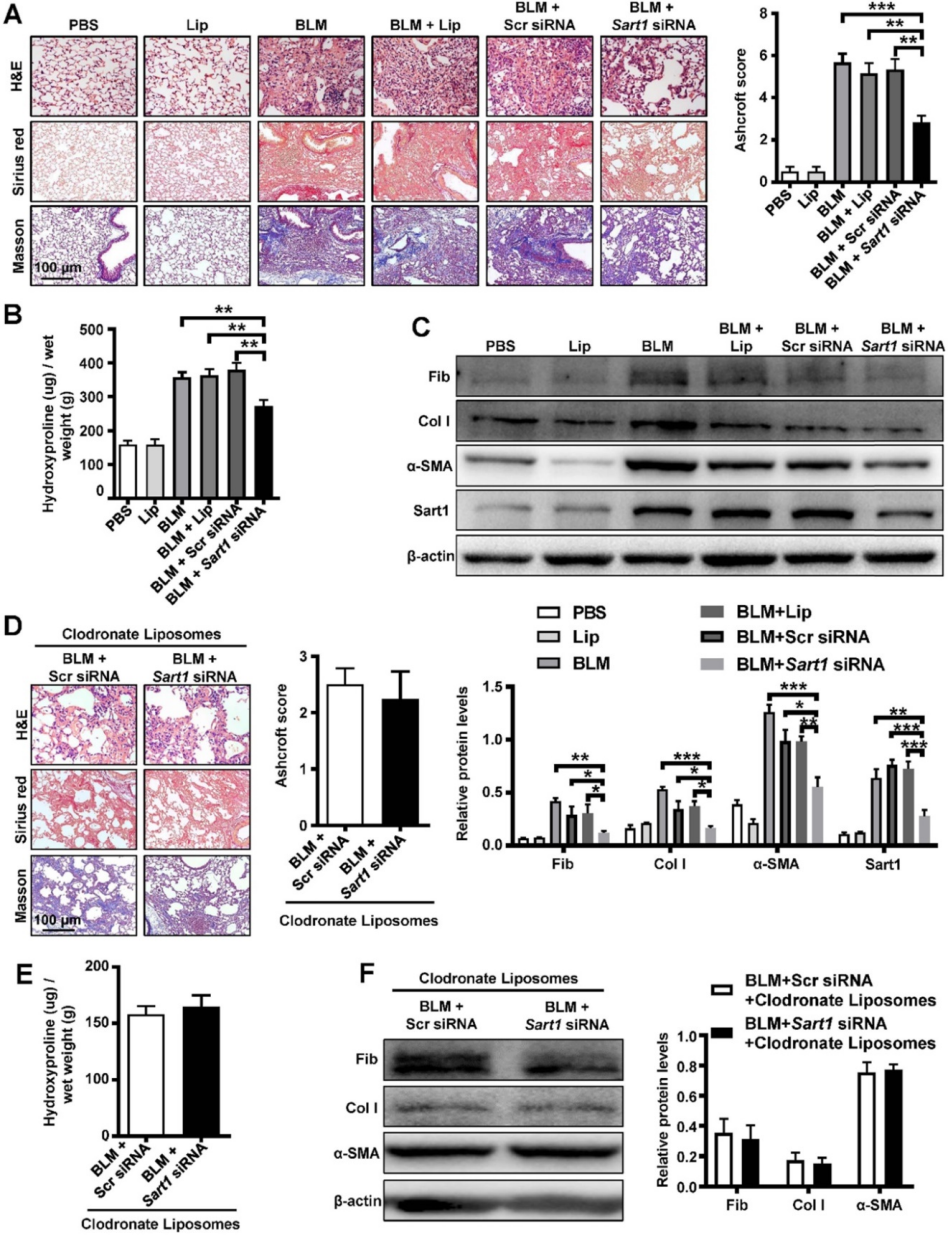
** Pulmonary administration of *Sart1* siRNA-loaded liposomes protected mice from BLM-induced lung injury and fibrosis. A:** Histological analysis of the severity of lung fibrosis in mice after BLM induction. Left panel: Representative results of H&E (top), Sirius red (center), and Masson (bottom) staining. Right panel: Bar graph showing the semiquantitative Ashcroft scores indicating the severity of fibrosis. Images were taken at an original magnification of ×200. **B:** Bar graph showing quantification of the hydroxyproline content in the lungs of mice after BLM induction. **C:** Western blot analysis of fibronectin, collagen I, α-SMA and Sart1 expression in lungs. Up panel: Representative results of Western blot. Down panel: Bar graph showing the mean data from all mice analyzed in each group. **D:** Depletion of macrophages abrogated the therapeutic effects of silencing *Sart1* expression on pulmonary fibrosis. Left panel: Representative results of H&E, Sirius red, and Masson staining. Images were taken at ×200 magnification. The right panel displays the semiquantitative Ashcroft scores, indicating the severity of fibrosis. **E:** Bar graph showing quantification of the hydroxyproline content in the lungs of mice. **F:** Western blot analysis of expression of fibronectin, collagen I, and α-SMA. Each bar represents the mean ± SEM of 6 mice analyzed. *P < 0.05; **P < 0.01; ***P < 0.001. BLM: bleomycin; Lip: Liposomes; Scr: Scramble; Fib: Fibronectin; Col I: Collagen I; α-SMA: α-smooth muscle actin.

Since IPF patients and mice were characterized by the overexpression of Sart1 in macrophages, we next sought to assess the therapeutic effect of *Sart1* siRNA on pulmonary fibrosis model by employing *Sart1* siRNA-loaded liposomes. Obvious and severe lung injury (H&E staining) and aberrant collagen accumulation (Sirius red and Masson staining) were observed in the BLM-treated group, liposomes-treated group and scramble siRNA-loaded liposomes-treated group at 21 days after BLM induction (Figure 5A), indicating serious pulmonary fibrosis. In contrast, in *Sart1* siRNA-loaded liposomes-treated mice, lung injury and fibrosis were significantly attenuated, which was accompanied by decreased collagen deposition (Figure 5A). Additionally, the *Sart1* siRNA-loaded liposomes-treated group achieved a lower Ashcroft score (Figure 5A, right panel), indicating relief from pulmonary fibrosis. Furthermore, the content of hydroxyproline, a marker correlated with fibrosis severity, was also measured in the lung homogenates. Indeed, the levels of hydroxyproline were much lower in the *Sart1* siRNA-loaded liposomes-treated group (Figure 5B). Consistently, Western blot analyses also revealed decreased expression of fibronectin, collagen I and α-SMA in *Sart1* siRNA-loaded liposomes-treated mice (Figure 5C). Consistently, the suppression of Sart1 in the lung by *Sart1* siRNA-loaded liposomes was corroborated *via* Western blot analysis (Figure 5C).

To address the assumption that the therapeutic effects of *Sart1* siRNA-loaded liposomes on pulmonary fibrosis depend on the induction of M2 macrophages, we first depleted lung macrophages by intratracheal injection of clodronate liposomes and then treated these mice with scramble siRNA-loaded liposomes or *Sart1* siRNA-loaded liposomes. Interestingly, we noted that *Sart1* siRNA-loaded liposomes-treated mice displayed a disease severity comparable to that of scramble siRNA-loaded liposomes-treated mice, as evidenced by histopathological analysis (Figure 5D) and fibrotic markers levels (Figure 5E-F), indicating that the protective effect of Sart1 knockdown is dependent on macrophages.

Collectively, our data suggest that suppression of Sart1 by the liposomes could protect mice against BLM-induced lung injury and fibrosis.

### Silencing of Sart1 attenuated M2 polarization *in vivo*

**Figure 6:**
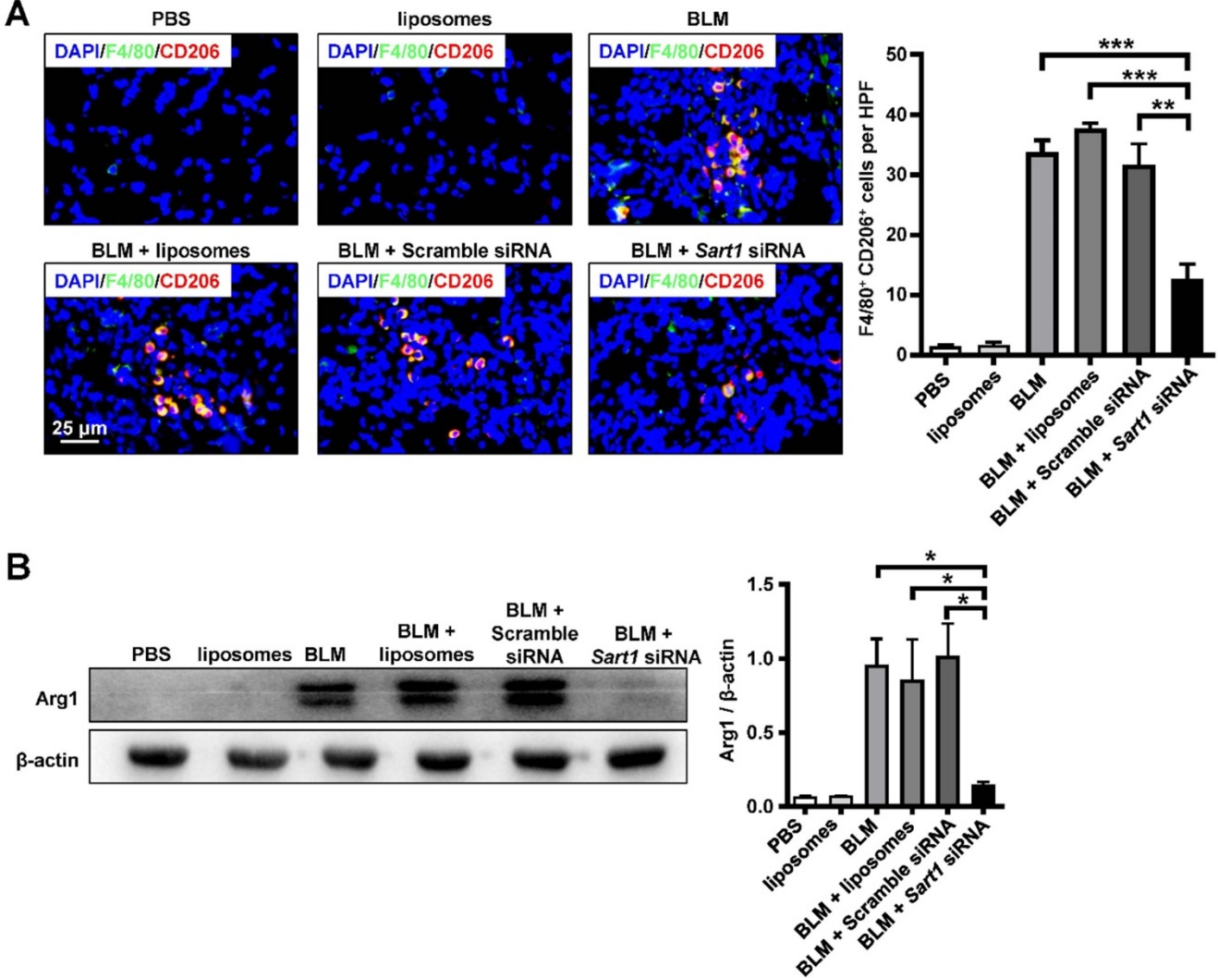
** Sart1 promoted the macrophage M2 program in BLM-induced mice. A:** Results of coimmunostaining for F4/80 (green) and CD206 (red). **B:** Arg1 expression in lung homogenates. Left panel: Representative results of Western blot. Right panel: Bar graph showing the expression levels of Arg1 in all mice of each group examined. Each bar represents the mean ± SEM of 6 mice analyzed. *P < 0.05; **P < 0.01; ***P < 0.001. Arg1: Arginase-1.

Since the *in vitro* experiment demonstrated that the liposomes could attenuate M2 macrophage polarization (Figure 3), we aimed to investigate whether the liposomes would exert similar effects *in vivo*. CD206 was employed as a marker of M2 macrophages for immunofluorescence staining (red). Indeed, in contrast with that in the BLM-treated group, liposomes-treated group and scramble siRNA-loaded liposomes-treated group, the number of M2 macrophages (F4/80^+^ CD206^+^ cells) was much lower in *Sart1* siRNA-loaded liposomes-treated group (Figure 6A), suggesting that the liposomes could modulate macrophage polarization *in vivo*. Moreover, Western blotting was conducted to verify the effect of the liposomes. Arginase-1 expression in lung homogenates from *Sart1* siRNA-treated mice was significantly decreased to one-seventh that of the scramble siRNA-loaded liposomes-treated group (Figure 6B), indicating a reduction in M2 macrophages in the lung. Taken together, our data verify that the liposomes could inhibit pulmonary macrophage polarization to the M2 phenotype with high efficiency during the pathological process of pulmonary fibrosis.

## Discussion

IPF is a chronic, progressive, fatal fibrosing interstitial lung disease of unknown etiology. The high mortality of IPF, together with the lack of available therapies for patients with end-stage disease, highlights the demand for better and applicable therapeutic approaches 35. Herein, we demonstrated that SART1 was mainly overexpressed in macrophages in the lungs of IPF patients and pulmonary fibrosis model mice. Importantly, *Sart1* siRNA-loaded liposomes could efficiently reduce the expression of Sart1 and suppress M2 macrophage polarization both *in vitro* and *in vivo*. The underlying mechanism was revealed to be the repression of Sart1, blocking the STAT6/PPAR-γ signaling pathway, which is pivotal for M2 macrophage polarization. Surprisingly, the liposomes selectively accumulated in the fibrotic area and were efficiently engulfed by pulmonary macrophages after intratracheal injection. As expected, administration of the *Sart1* siRNA-loaded liposomes dramatically improved BLM-induced lung injury and fibrosis, as manifested by a reduction in the histopathological score and decreased expression of the fibrotic proteins, along with attenuated M2 macrophage infiltration in the lung.

Sart1 is an isoform of a specific E3 ubiquitin ligase that is expressed in malignant proliferating tissue and has been recognized as involved in spliceosome assembly and cell division 19. Sart1 specifically degrades HIF-1α in an oxygen-independent manner 18. Notably, the absence of HIF-1α may activate macrophages to the M2 program 20. We further noted that macrophages originating from the lungs of patients with IPF and pulmonary fibrosis model mice exhibited significantly upregulated Sart1 expression. These observations prompted us to hypothesize that Sart1 modulated the M2 macrophage program. To further address the impact of Sart1 on macrophage polarization, BMDMs were cultured and transfected with *Sart1* siRNA-loaded liposomes, followed by IL-4 or LPS treatment. In line with our expectations, Western blot and RT-PCR analyses revealed that silencing Sart1 significantly inhibited the IL-4-induced production of M2 macrophages, as corroborated by the reduced expression of Arg-1,* CD206*, *Ym1* and *Fizz1*. Additionally, knockdown of Sart1 seemed to increase M1 macrophage polarization, as manifested by increased expression of *iNOS*, a marker of M1 macrophages.

Since this is the first study to validate that Sart1 can modulate macrophage polarization, we attempted to unmask the underlying mechanism. Previous studies have demonstrated that the STAT6/PPAR-γ signaling pathway is essential for macrophage polarization 32, 36. Therefore, the expression levels of p-STAT6 and PPAR-γ were evaluated in gain and loss-of-function assays. As shown by Western blot analysis, silencing *Sart1* markedly suppressed the expression of p-STAT6 and PPAR-γ. While opposing results were observed in Sart1 overexpressed BMDMs. Furthermore, inhibitors of p-STAT6 and PPAR-γ could significantly abrogate M2 macrophages program induced by pCMV-*Sart1*, indicating that Sart1 induced macrophage adoption of the M2 phenotype by activating the STAT6/PPAR-γ signaling pathway.

In the human genome, different types of genes regulate cell growth and function in a precise way 37. Abnormal expression of genes over time causes the occurrence and development of various diseases. The discovery of RNA interference (RNAi) in mammalian cells provided a new perspective for gene therapy as an alternative approach for disease therapeutics 38. siRNAs can silence the expression of almost any gene with high efficiency and specificity, including targets previously considered to be “undruggable”. However, the security of a vector for siRNA delivery is a key point in realizing the broad potential of siRNA-based therapeutics. As previous reports have indicated 39, siRNA molecules are too large and hydrophilic to penetrate cell membranes alone. In addition, their high negative charge and stiffness make siRNA unstable in serum 40. Hence, in this study, we employed a type of nanoparticle, liposomes, to address the above issues. The chosen liposomes could be simply prepared and conveniently transformed for clinical application 41. Nanoparticles show excellent biocompatibility and a high encapsulation efficiency, along with reduced gene degradation and enhanced targeting capability compared to those of traditional gene therapy 42. The cationic nature of liposomes contributes to their efficient encapsulation of negatively charged siRNA 14. Remarkably, the favorable biocompatibility of liposomes leads to much lower immune responses and reduced cytotoxicity compared to those upon administration of viral or bacterial-based vectors 43, 44.

One of the basic functionalities of macrophages is their elimination of foreign substances, resulting in the massive phagocytosis of nanoparticles by macrophages 15. Generally, to increase therapeutic efficacy and decrease side effects, multiple strategies, such as PEGylation and HESylation, have been adopted to assist nanovehicles in evading nonspecific uptake by macrophages 45, 46. However, the results have been unsatisfactory 47. In this investigation, in contrast, we exploited only the phagocytic property of macrophages. Since a mass of macrophages were recruited into the fibrotic area during the pathogenesis of pulmonary fibrosis, the intratracheal-injected liposomes were effectively engulfed by the recruited macrophages and concentrated mainly in the fibrotic area. This finding further corroborated that liposomes are quite suitable for the treatment of pulmonary fibrosis by their targeting of macrophages. Moreover, we adopted intratracheal injection as an administration route rather than systemic administration, which exhibits better specificity and an improved targeting effect for pulmonary diseases 48, 49. Intratracheal administration, which is equivalent to clinical atomization treatment, minimized the unexpected uptake of liposomes by the reticuloendothelial system (RES, mainly by the liver or spleen), as shown in Figure 4B. Therefore, *via* intratracheal injection, the liposomes could achieve enhanced targeting capability and minimized side effects for the treatment of pulmonary fibrosis, making their clinical transformation easy.

Macrophages have been implicated in the pathogenesis of the profibrotic response 30, 50. An enhanced M2 macrophage program is considered a prominent mechanism associated with the fibrotic remodeling of organs, including the lung. Previous studies, including ours, have demonstrated that inhibition of macrophage polarization to the M2 phenotype could be a potential strategy for IPF 31, 51. Interestingly, we found that suppression of Sart1 could blunt the macrophage M2 program *in vivo*, as evidenced by the decreased number of F4/80^+^CD206^+^ cells in the lungs of mice treated with *Sart1* siRNA-loaded liposomes. This result was further corroborated by Western blot analyses of lung homogenates. In summary, our data suggest that suppressing Sart1 by siRNA-loaded liposomes could be a promising strategy for the treatment of pulmonary fibrosis in clinical settings.

## Conclusion

The results of this investigation showed that Sart1 plays a critical role in IPF by promoting M2 macrophage polarization through activation of the STAT6/PPAR-γ signaling pathway. In addition, *Sart1* siRNA-loaded liposomes administered *via* intratracheal injection could effectively target pulmonary fibrotic areas and greatly protected mice from BLM-induced pulmonary fibrosis. This study suggests that *Sart1* siRNA-loaded liposomes may be a promising therapeutic strategy for pulmonary fibrosis in clinical settings.

## Supplementary Material

Supplementary figures.

## Figures and Tables

**Table 1:** Characteristics of subjects from which lung samples were taken

	Lung samples		*P* value
	IPF (n = 8)	Control (n = 6)	
Age (years)	58.50 ± 3.645	55.17 ± 6.024	0.6265
Smoking index	41.10 ± 19.15	46.58 ±24.90	0.8620
BMI	22.18 ± 1.170	23.40 ± 0.3360	0.3966
**Sex**			0.8860
Female	3 (37.50%)	2 (33.33%)	
Male	5 (62.50%)	4 (66.67%)	
**FVC**			
Percent Predicted	65.04 ± 4.565	NA	
DLCO	42.10 ± 4.011	NA	

BMI: body mass index; FVC: forced vital capacity; DLCO: diffusion capacity for carbon monoxide.

## Abbreviations

Arg1: Arginase-1
α-SMA: α-smooth muscle actin
BLM: bleomycin
BMDMs: bone marrow-derived macrophages
BMI: body mass index
Col I: collagen I
DLCO: diffusion capacity for carbon monoxide
DSL: dynamic light scattering
Fib: fibronectin
Fizz1: Found in inflammatory zone 1
FVC: forced vital capacity
HPF: high-power field
iNOS: inducible nitric oxide synthase
IPF: idiopathic pulmonary fibrosis
M-CSF: macrophage colony-stimulating factor
NSCLC: non-small cell lung cancer
PDI: polymer dispersity index
PPAR-γ: Peroxisome proliferator-activated receptor γ
SART1: spliceosome associated factor 1
STAT6: Signal transducer and activator of transcription 6
TEM: transmission electron microscopy
