# Supplementary material for: Suppressing Sart1 to modulate macrophage polarization by siRNA-loaded liposomes: a promising therapeutic strategy for pulmonary fibrosis
Source: Theranostics. 2021 Jan 1;11(3):1192–206. doi: 10.7150/thno.48152 (PMC7738894; doi:10.7150/thno.48152)
Supplement: Supplementary file 1 — Supplementary figures. [file thnov11p1192s1.pdf]

Supplementary figure

Figure S1: Results of coimmunostaining for F4/80 (red) and CD206 (green) in saline-treated or BLM-induced mice. The images were taken at ×200 magnification. Each bar represents the mean ± SEM of 6 mice analyzed. ***P < 0.001.

Figure S2: A: The *in vitro* biocompatibility of siRNA-loaded liposomes was evaluated by CCK8 assay. **B:** Western blot analysis of the transfection efficacy of siRNA targeting Sart1 in liposomes transfected in RAW264.7 cells with Lipofectamine 3000. Upper panel: Representative Western blot. Down panel: Figures showing the data from 3 replicates. **P < 0.01.

Figure S3: Overexpression of Sart1 in BMDMs after pCMV-*Sart1* transfection. Left panel: Representative Western blot result. Right panel: Figures showing the data with from 3 replicates. ***P < 0.001.

Figure S4: Immunofluorescence image for showing the biodistribution of DiR-labelled scramble siRNA-loaded liposomes and *Sart1* siRNA-loaded liposomes in the lungs from of BLM-induced mice.

Figure S5: Histological analysis of the lung tissues of mice after pulmonary administration of saline or liposomes. Images were captured at ×200 magnification.

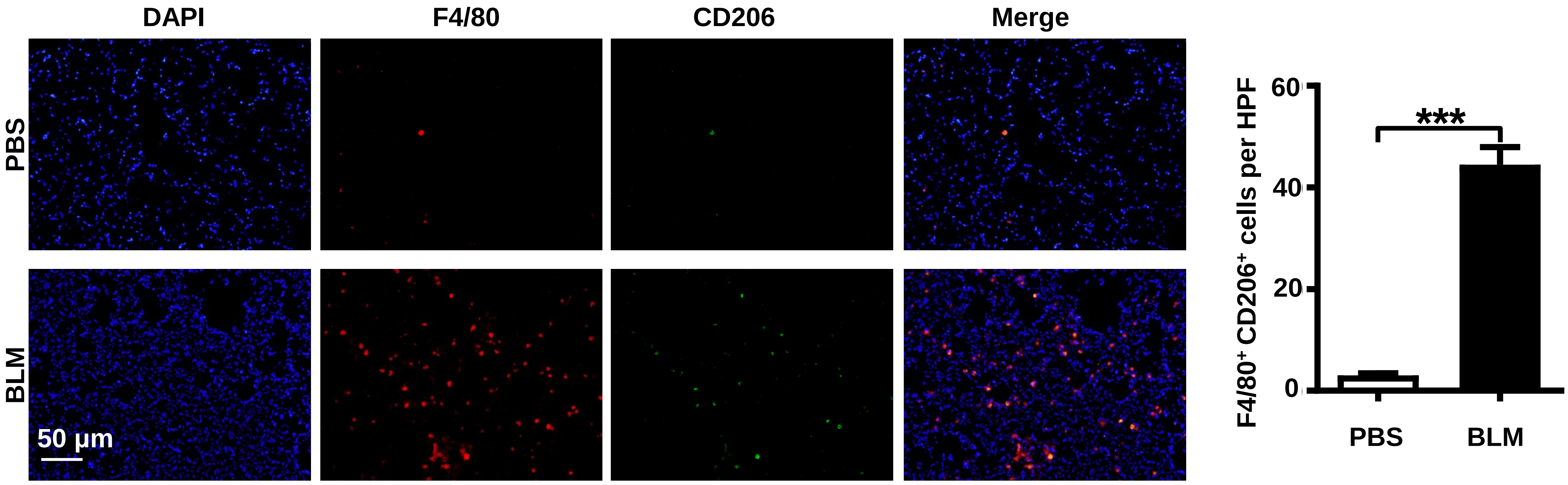
DAPI
F4/80
CD206
Merge
PBS
BLM
50 μm
F4/80+ CD206+ cells per HPF
60
40
20
0
***
PBS
BLM

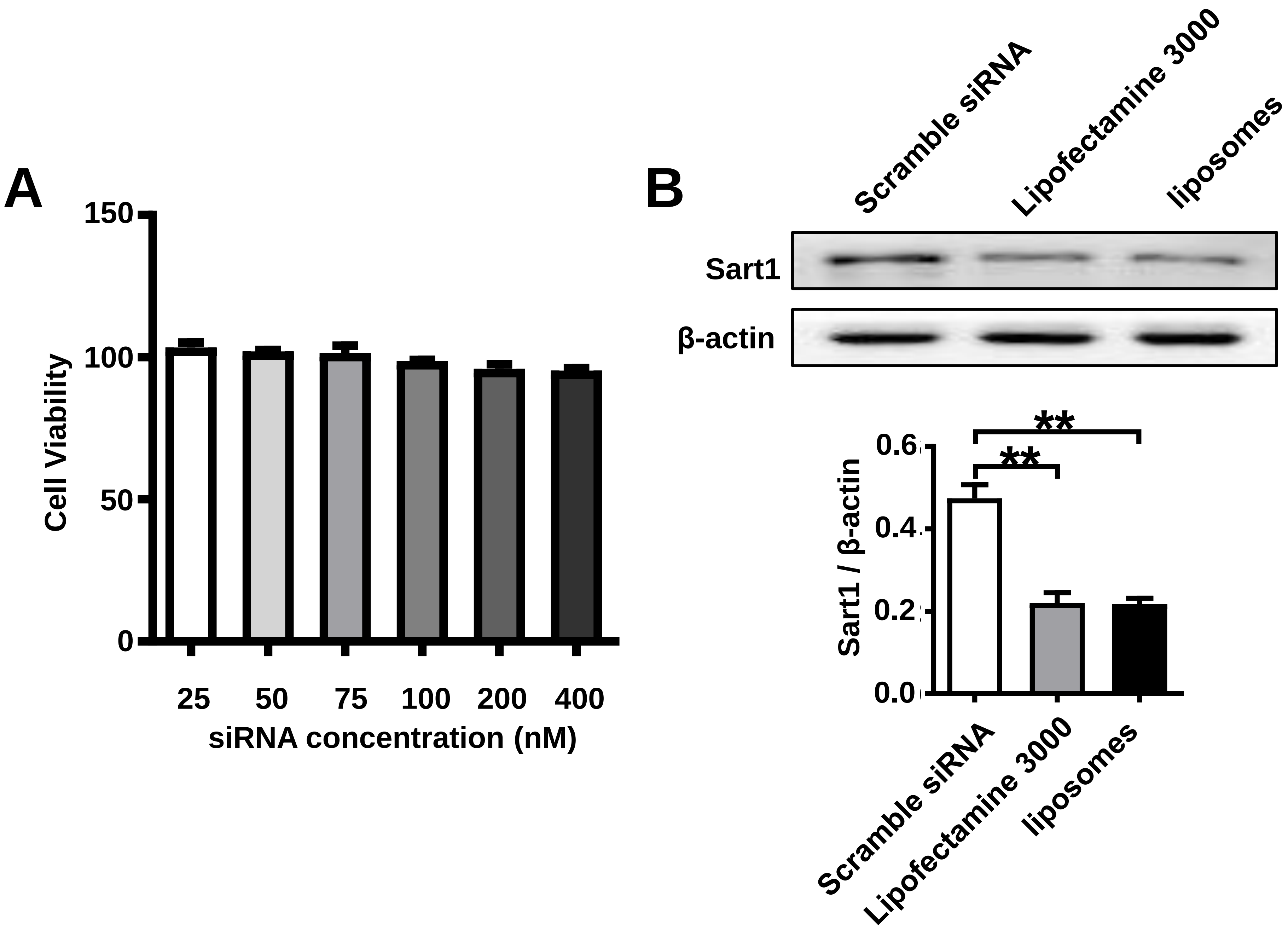
A
150
100
50
0
Cell Viability
25
50
75
100
200
400
siRNA concentration (nM)
B
Scramble siRNA
Lipofectamine 3000
liposomes
Sart1
β-actin
**
**
0.6
0.4
0.2
0.0
Sart1 / β-actin
Scramble siRNA
Lipofectamine 3000
liposomes

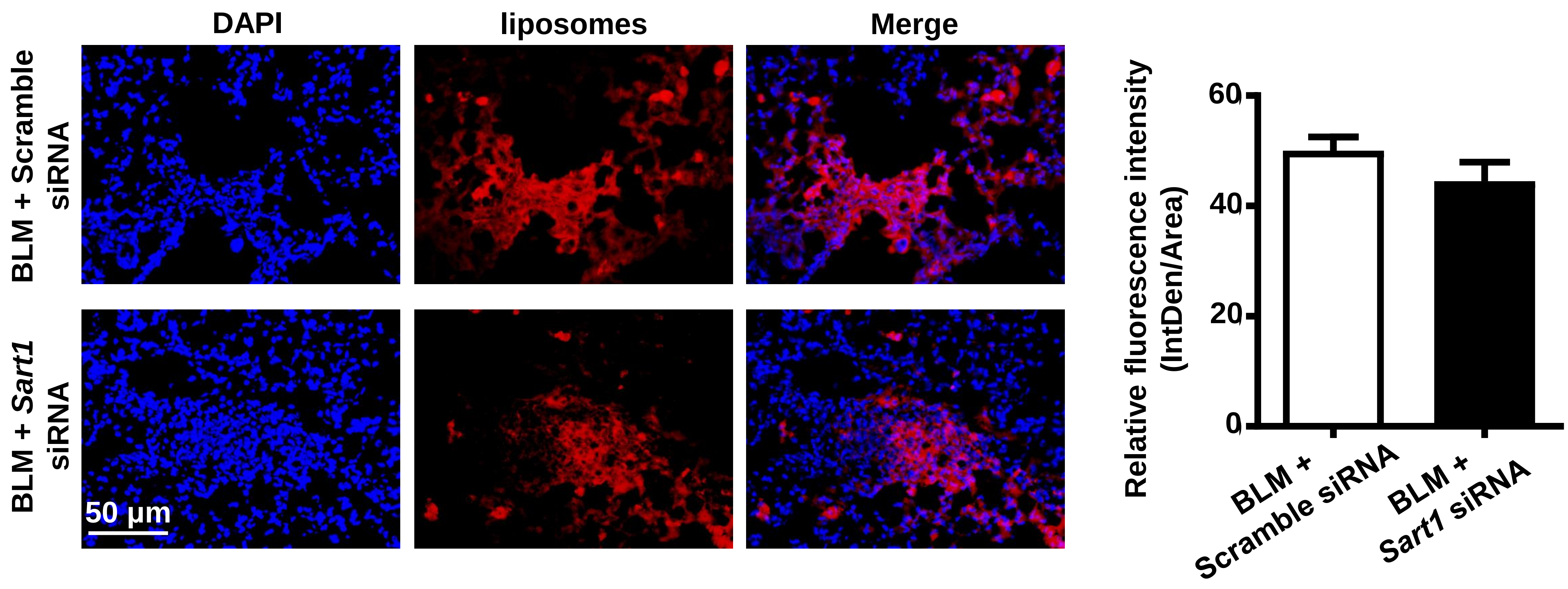
DAPI
liposomes
Merge
BLM + Scramble siRNA
BLM + Sart1 siRNA
50 µm
Relative fluorescence intensity (IntDen/Area)
60
40
20
0
BLM + Scramble siRNA
BLM + Sart1 siRNA

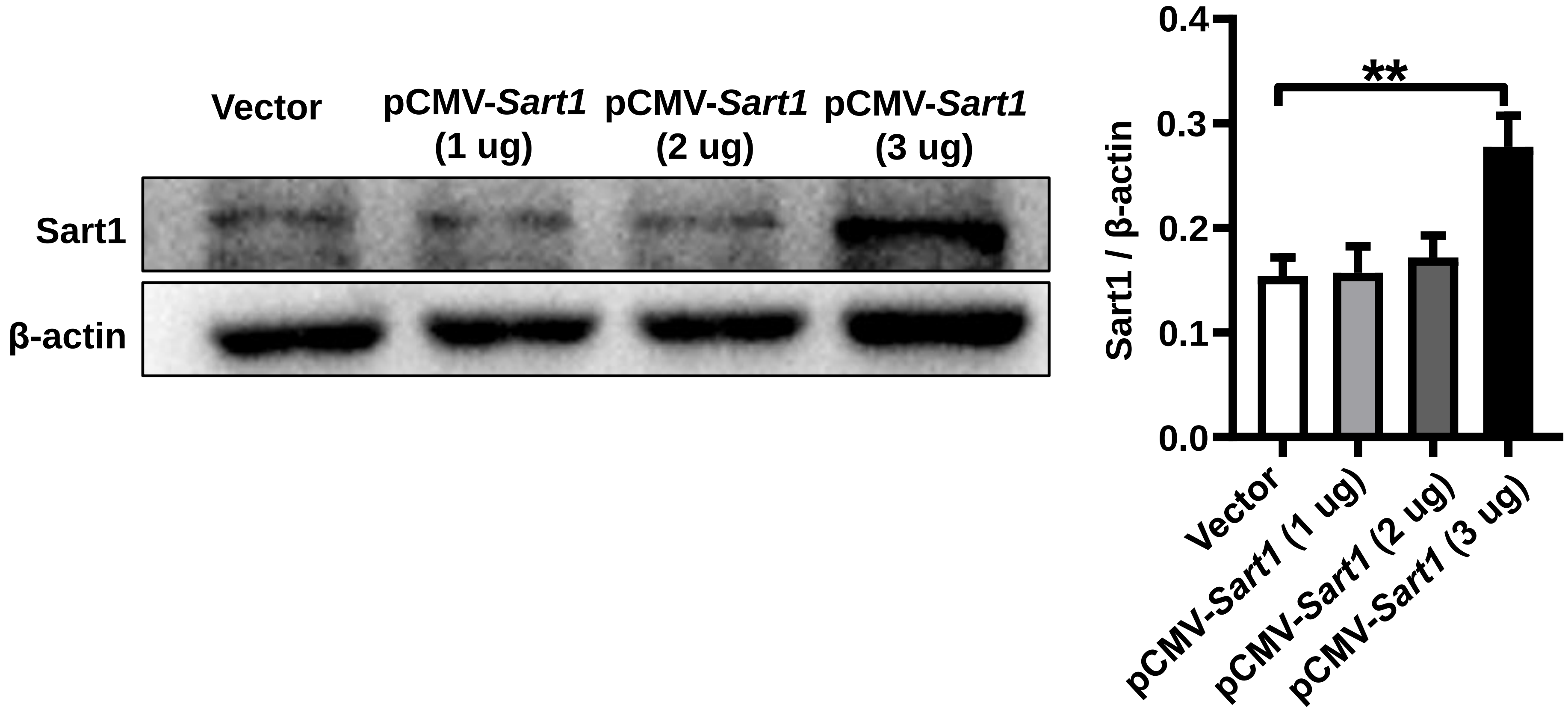
Vector
pCMV-Sart1 (1 ug)
pCMV-Sart1 (2 ug)
pCMV-Sart1 (3 ug)
Sart1
β-actin
Sart1 / β-actin
0.0
0.1
0.2
0.3
0.4
**
Vector
pCMV-Sart1 (1 ug)
pCMV-Sart1 (2 ug)
pCMV-Sart1 (3 ug)

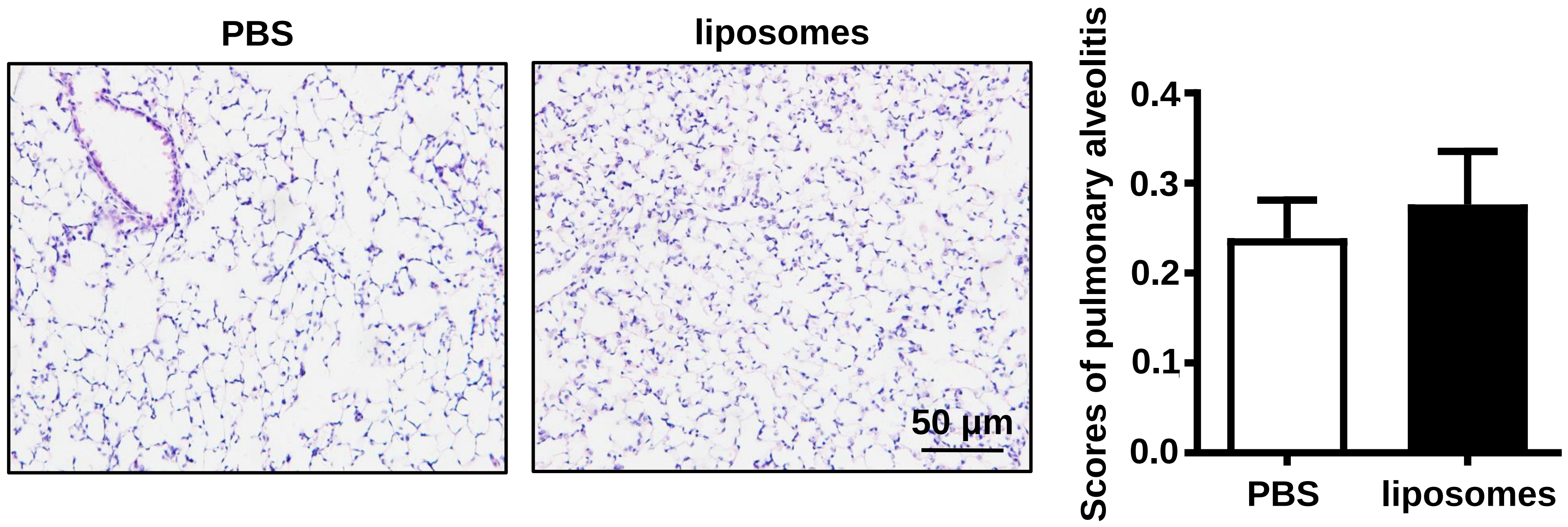
PBS
liposomes
50 μm
Scores of pulmonary alveolitis
0.4
0.3
0.2
0.1
0.0
PBS
liposomes
